# Oral manifestations of hereditary nonpolyposis colorectal cancer syndrome: a family case series

**DOI:** 10.1186/1752-1947-8-249

**Published:** 2014-07-10

**Authors:** Fabiana Tolentino Almeida, Raquel Ribeiro Gomes, André Ferreira Leite, João Batista Sousa, Ana Carolina Acevedo, Eliete Neves Silva Guerra

**Affiliations:** 1 Oral Care Center for Inherited Diseases, Division of Dentistry at University Hospital of Brasilia, Faculty of Health Sciences, University of Brasilia (UNB), Brasilia, Brazil; 2 Coloproctology Division, University Hospital of Brasilia, Faculty of Medicine, University of Brasilia (UNB), Brasilia, Brazil

**Keywords:** Dento-osseous anomalies, Fordyce granules, Hereditary nonpolyposis colorectal cancer

## Abstract

**Introduction:**

Hereditary nonpolyposis colorectal cancer is a colorectal cancer syndrome characterized by the development of colorectal cancer and extracolonic tumors, and this syndrome has an autosomal dominant mode of inheritance. To our knowledge, our study was the first to find dento-osseous anomalies and the second to observe Fordyce granules in a family with individuals with hereditary nonpolyposis colorectal cancer.

**Case presentations:**

Twenty members of one Brazilian family with individuals with hereditary nonpolyposis colorectal cancer were analyzed according to the presence of colorectal cancer and the occurrence of Fordyce granules and dento-osseous anomalies. Their average age was 29.6 (range 7 to 53 years) years. Medical examinations of this family with hereditary nonpolyposis colorectal cancer were performed at the Coloproctology Division of our hospital. Then, all individuals were referred to our Oral Care Center for Inherited Diseases for intraoral examinations to verify the presence of Fordyce granules. Dental panoramic radiographs were done in order to describe dento-osseous anomalies on applying the Dental Panoramic Radiograph System. Of the 20 family members, four were diagnosed with hereditary nonpolyposis colorectal cancer and all four presented Fordyce granules in their upper lip, but only one of these four patients (Case 2) had a significant dento-osseous anomaly.

**Conclusions:**

Our familial study verified the presence of Fordyce granules in all individuals diagnosed with hereditary nonpolyposis colorectal cancer, and the presence of significant dento-osseous anomalies in one of these cases. However, the relationship between oral manifestations and hereditary nonpolyposis colorectal cancer should be further investigated.

## Introduction

Colorectal cancer (CRC) is the third most common tumor in developed countries [1,2]. In Brazil, it is among the five more common malignant neoplasias and it is the third in mortality in both sexes [3]. Hereditary nonpolyposis colorectal cancer (HNPCC) or Lynch syndrome (Lynch syndrome, Online Mendelian Inheritance in Man database number 120435) is the most common form of hereditary CRC; it has an autosomal dominant mode of inheritance [1]. HNPCC has a high penetrance caused by a germline mutation in mismatch repair genes, most frequently *MLH1* or *MSH2*. In order to establish the diagnosis of HNPCC, families are analyzed and classified according to Amsterdam Criteria I and II [4,5]. The disease is characterized by the development of CRC at an early age (approximately 45-years old), which is located in the proximal colon in two-thirds of cases, and occurrence of extracolonic tumors (ovaries, endometrium, stomach and other) in affected families [5-7]. In a Brazilian HNPCC familial study, breast cancer followed by endometrial and uterine cervix cancer were the most frequent extracolon cancers found in women, and prostate and stomach tumors in men [8]. Early identification of families and individuals at high risk is essential to reduce CRC incidence. Furthermore, disease morbidity and mortality can be reduced if individuals are diagnosed and tumors are removed in time [7].

Oral ectopic sebaceous glands, known as Fordyce granules (FGs), and alteration in vascular patterns in the oral mucosa are reported changes regarding oral manifestations of HNPCC [9-11]. An increase in number and size of sebaceous glands following activation of the hedgehog pathway has been reported [12]. This signaling pathway plays multiple roles in animal development and has been linked to the development and progression of several forms of cancer [13,14]. FGs are common in adult patients, and are benign lesions that can appear in healthy individuals [15]. Epidemiological studies have demonstrated the presence of FGs in 3.8% to 27.9% [16-19] of the normal population with a higher prevalence in male patients [16,17]. However, it has been suggested that FGs may be associated with systemic disease and clinical observation could be helpful to increase the rate of HNPCC diagnosis in affected families [10]. To date, only a few studies have tested the association between HNPCC and oral manifestations [12-14].

Dento-osseous anomalies such as osteomas, dense bone island, odontomas, and supernumerary and impacted teeth are also proliferative benign lesions related to dento-osseous development and they are well documented in familial adenomatous polyposis (FAP) [20,21]. To our knowledge, dento-osseous alterations have not been previously reported in individuals with HNPCC. A weighted scoring system to evaluate dento-osseous anomalies known as Dental Panoramic Radiograph Score (DPRS) was proposed by some authors to diagnose FAP. In their study, the test took into consideration the nature, extent, and site of osseous and dental changes on dental panoramic radiographs [22]. The significance of the findings was determined by assigning a specific range of values of DPRS. With a DPRS≥7, the specificity and positive predictive rate was 100%, and a false positive rate of 0% was obtained [22]. Therefore, DPRS was found to be a reproducible and valid index for assessing the diagnostic significance of dento-osseous changes in individuals at 50% risk of FAP, even when performed by relatively inexperienced examiners [23]. This scoring system has never been tested in individuals with HNPCC.

Establishing the diagnosis for HNPCC poses a challenge and requires knowledge and vigilance [24]. The study of oral findings might be helpful in the development of a noninvasive and inexpensive test for recognizing individuals with HNPCC. Thus, the purpose of the present study was to verify oral manifestations and to investigate dento-osseous anomalies applying the DPRS system in a family case series diagnosed with HNPCC.

## Case presentations

Twenty members of one family with HNPCC (11 men and 9 women) belonging to three generations were examined. The study was approved by the Ethics Committee (number: 099/2008) of our institution. A written informed consent was obtained from all examined family members. The individuals were analyzed according to the presence of CRC and the occurrence of FGs and dento-osseous anomalies. Their average age was 29.6 (range 7 to 53 years) years. Firstly, the diagnosis of the case index was established. Then, the other 19 members of the family were examined by the Coloproctology Division of our hospital, in order to determine whether individuals presented HNPCC according to the Amsterdam Criteria I and II [5]. Afterwards, a questionnaire with personal data (age, sex), overall health and diagnosis of HNPCC, CRC and extracolon cancer was filled for each individual.

Then, all individuals were referred to our Oral Care Center for Inherited Diseases for intraoral examinations to verify the presence of FGs. Dental panoramic radiographs were done in order to describe dento-osseous anomalies on applying the Dental Panoramic Radiograph System. Their oral cavities were examined using artificial light and their oral mucosal surfaces were evaluated with a focus on the identification of FGs. Dental panoramic radiographs were taken from individuals with HNPCC and their relatives. The dento-osseous alterations were evaluated on dental panoramic radiographs using the DPRS, developed by Thakker *et al.*[22]. In this analysis, the following anomalies were evaluated: dense bone islands, hazy sclerosis, osteomas, odontomas, supernumerary teeth and unerupted teeth following the criteria proposed by the aforementioned authors [22]. Single scores were given to each dento-osseous alteration observed in the radiographs depending on the number and size of the anomaly. An overall DPRS was determined for each individual by the addition of each single score. The final score indicates one of four possible outcomes: normal (0 to 2), minimal changes (3 to 4), equivocal changes (5 to 6) and significant changes (≥7). The radiographs were evaluated blind to the HNPCC diagnosis and in consensus by three oral and maxillofacial radiologists on a flat viewing box under dimmed lighting.

**Figure 1:**
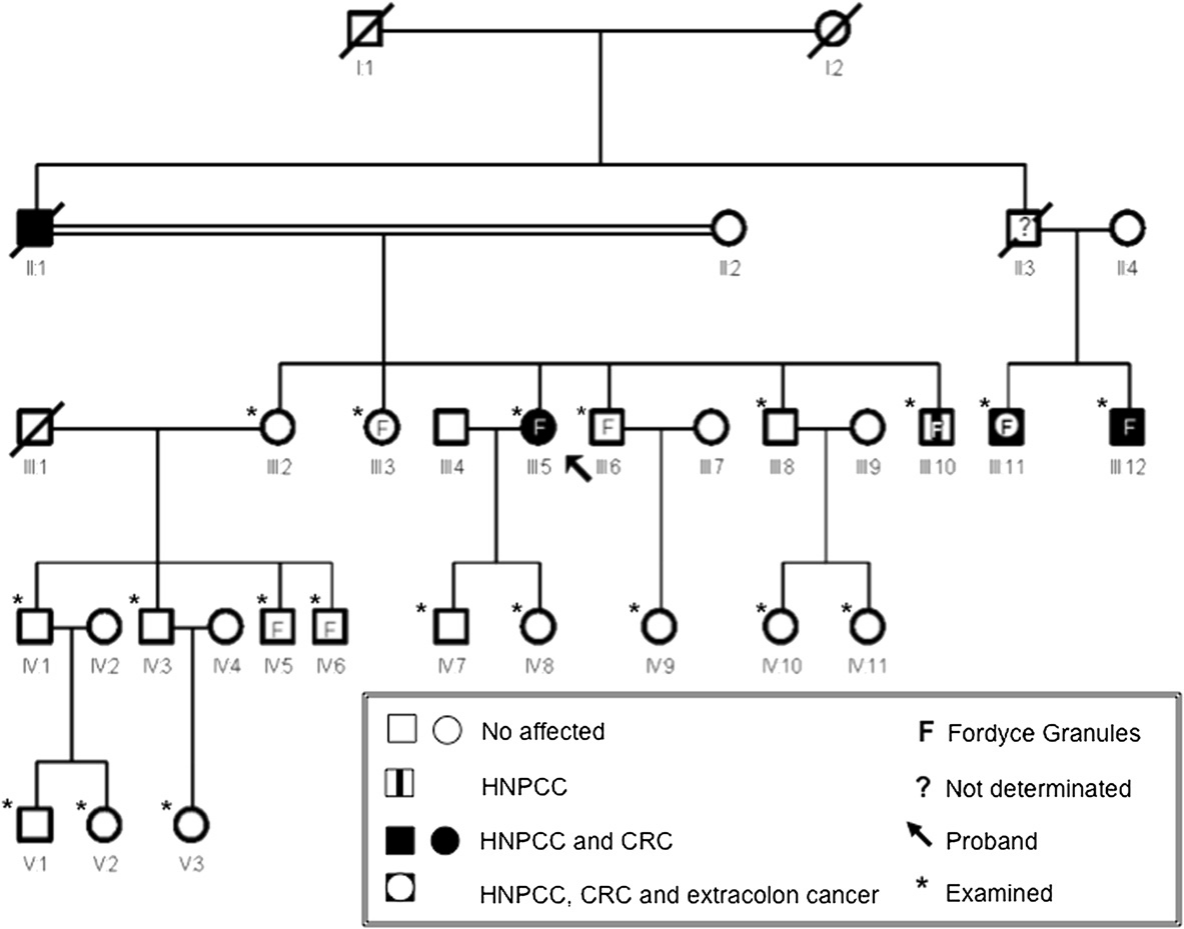
**Pedigree of the family.** Pedigree of the family with hereditary nonpolyposis colorectal cancer showing an autosomal dominant mode of inheritance that presents hereditary nonpolyposis colorectal cancer, colorectal cancer, extracolon cancer and Fordyce granules. In pedigree, squares represent males and circles represent females. The diagonal line in some squares and circles are used to indicated died patients. Abbreviations: CRC, colorectal cancer; HNPCC, hereditary nonpolyposis colorectal cancer.

**Figure 2:**
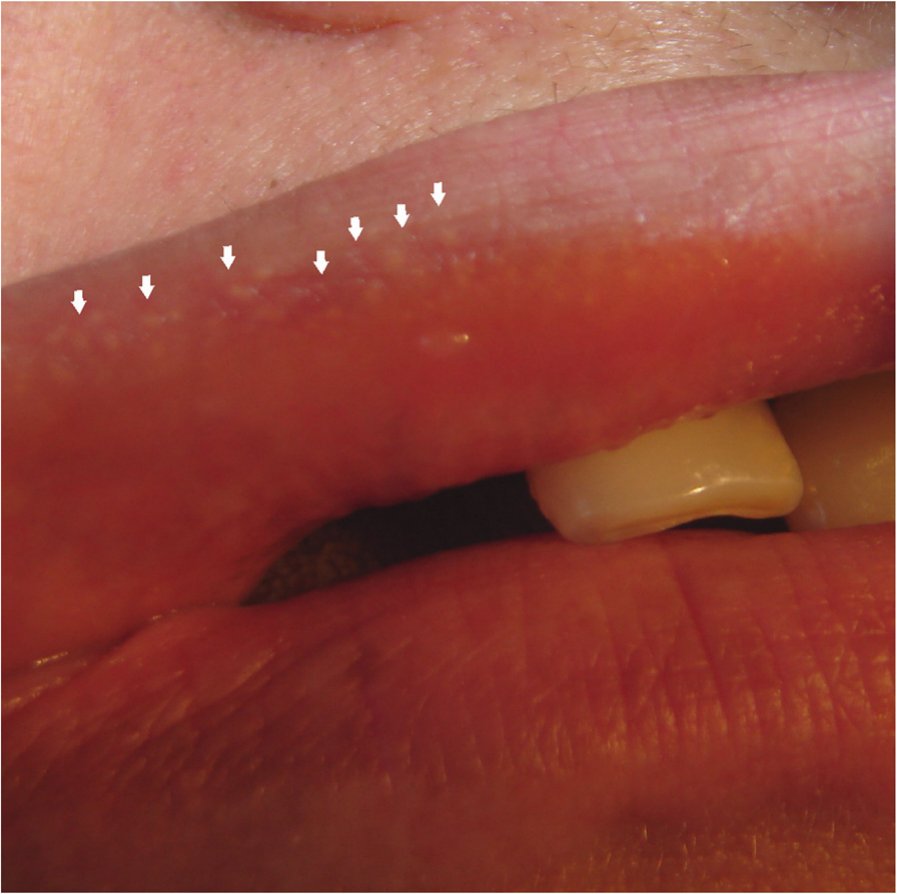
**Extraoral photograph.** Extraoral photograph image showing Fordyce granules in the upper lip (arrows) of individual III:5 with hereditary nonpolyposis colorectal cancer and colorectal cancer.

The pedigree of the studied family showed HNPCC inherited in an autosomal dominant mode (Figure 1). The main characteristics of all studied individuals are summarized in Table 1.Four individuals had the diagnosis of HNPCC. All of these patients presented FGs and one had a significant dento-osseous anomaly (hazy diffuse sclerosis), as follows below:Case 1 – A 49-year-old Brazilian woman (Case index, III:5, Figure 1) presented HNPCC and CRC. FGs were found in her upper lip (Figure 2). Dento-osseous anomalies were not found in the dental panoramic radiograph of this patient.

**Table 1:** Medical and oral findings in family members with hereditary nonpolyposis colorectal cancer

**Individuals in pedigree**	**Gender**	**Age (years)**	**CRC**	**Extracolon cancer**	**HNPCC**	**Fordyce granules**	**DPRS**
III:2	F	53	–	–	–	–	8
III:3	F	54	–	–	–	+	2
III:5	F	49	+	–	+	+	0
III:6	M	49	–	–	–	+	2
III:8	M	43	–	–	–	–	0
III:10	M	40	–	–	+	+	10
III:11	M	47	+	+	+	+	0
III:12	M	37	+	–	+	+	0
IV:1	M	34	–	–	–	–	0
IV:3	M	30	–	–	–	–	0
IV:5	M	24	–	–	–	+	0
IV:6	M	21	**–**	**–**	**–**	**+**	0
IV:7	M	18	–	–	–	–	0
IV:8	F	14	–	–	–	–	5
IV:9	F	12	–	–	–	–	0
IV:10	F	11	–	–	–	–	5
IV:11	F	10	–	–	–	–	0
V:1	M	15	–	–	–	–	0
V:2	F	13	–	–	–	–	0
V:3	F	07	–	–	–	–	0

*Abbreviations*: CRC colorectal cancer, DPRS Dental Panoramic Radiograph Score, F = female, HNPCC hereditary nonpolyposis colorectal cancer, M male, +, present; -, absent.

Case 2 – A 40-year-old Brazilian man (III:10) had the diagnosis of HNPCC. FGs were found only in his upper lip. The DPRS analysis showed a significant dento-osseous anomaly (≥7) due to a hazy diffuse sclerosis.

Case 3 – A 47-year-old Brazilian man (III:11) presented HNPCC, CRC and an extracolon associated tumor (hepatic cancer). FGs were found in his upper lip, and the DPRS analysis did not demonstrate dento-osseous anomalies.

Case 4 – A 37-year-old Brazilian man (III:12) had HNPCC and CRC, and the presence of FGs in his upper lip. Furthermore, no dento-osseous anomalies were found in the radiographic examination.

Other than these four individuals with HNPCC described above, 25% (4 individuals) of the unaffected family members also presented FGs. Three individuals had FGs located both in the upper lip and oral mucosa, and one had FGs only in the oral mucosa.

Regarding dento-osseous anomalies, besides the affected individual with HNPCC (III:10, Figure 1), an unaffected family member (III:2, Figure 1) also presented a significant DPRS due to a hazy diffuse sclerosis (Table 1).

## Discussion

In this study, 20 members of a Brazilian family with HNPCC were evaluated and oral examinations were performed with a focus on FGs and dento-osseous anomalies. HNPCC was diagnosed in four individuals and all four presented FGs in their upper lip whereas significant DPRS was observed only in one of these patients. Moreover, considering the whole sample (20 family members), FGs and significant DPRS were found in 40% and 10%, respectively.

De Felice *et al.*[10] published the first study that assessed a possible association between FGs in the oral mucosa and HNPCC. In their study, FGs were found to be approximately 50 to 90 times higher in individuals with HNPCC than in controls. They suggested that FGs could be a new phenotypic marker of families with HNPCC [10]. Our results are in agreement with their results and reinforce their proposition that clinical observation of FGs could increase the rate of HNPCC diagnosis in affected families. In our study, all individuals with HNPCC presented FGs, whereas FGs were observed in 25% of the unaffected family members. Our percentage of unaffected family members with FGs is slightly higher than the 20.9% frequency reported in a previous Brazilian study [19], although our results may be underestimated given that 50% of the unaffected individuals are children or teenagers. Considering only post-puberty family members, the frequency of FGs increased to 50%; it is higher than the prevalence previously reported in general individuals (Table 2) [16-19]. Because 25% of the unaffected family members also presented FGs (and 50% of the adults), this oral manifestation cannot still be considered a pathognomonic feature of HNPCC.

**Table 2:** Frequency of Fordyce granules in general population and in individuals with hereditary nonpolyposis colorectal cancer

**Author (year)**	**Samples**	**Number of individuals**	**Frequency of Fordyce granules (%)**
**Type of study**			
Reichart [16]	General population (Germany)	2022	26.6% (group 1: 35–44 years)
Cross-sectional study			23.7% (group 2: 65–74 years)
Jahanbani *et al*. [17]	General population (Iran)	598	27.9%
Cross-sectional study			
Al-Mobeeriek and AlDosari [18]	General population (Saudi Arabia)	2552	3.8%
Cross-sectional study			
Ferreira *et al*. [19]	General population (Brazil)	335	20.9%
Cross-sectional study			
De Felice *et al.*[10]	Individuals with HNPCC and controls (Italy)	15 (HNPCC)	86.7% of the cases
Case–control study		630 (controls)	0.95% of the controls
Present study**,** 2010–2011	Individuals with HNPCC and relatives (Brazil)	20 (one family with HNPCC)	40% all of the sample
Familial study			100% (individuals with HNPCC)
			25% (unaffected individuals)

*Abbreviations*: HNPCC hereditary nonpolyposis colorectal cancer.

Dento-osseous anomalies associated with FAP disease are well reported in the literature [20]; however, they have not been related to individuals with HNPCC. Significant dento-osseous anomalies were observed in two family members, although only one had HNPCC diagnosis. Four other individuals without HNPCC presented minimal dento-osseous anomalies, nevertheless these results may be due to the amount of young individuals in this family, and some dento-osseous anomalies may have an age-dependent appearance. A previous study suggested that a DPRS test is more likely to be informative when performed in an older group of individuals [22]. Therefore, follow-up of the youngest individuals is recommended. Dento-osseous anomalies may be found in the general population. However, the lack of data in the literature regarding the prevalence of these dento-osseous anomalies precluded a direct comparison with our results.

It is important to draw attention to the relationship between CRC and oral findings. In our study, all individuals who had CRC presented FGs and DPRS equal to zero. In contrast, individuals with significant DPRS did not have CRC, yet one had HNPCC. On the one hand, this could suggest that individuals with FGs have higher risk to develop CRC, on the other hand, individuals with significant DPRS have a lower risk. More studies with a larger number of individuals and families are necessary though to confirm this proposition.

## Conclusions

In summary, in the present familial study, FGs were observed as a feature in all individuals with HNPCC diagnosis. In addition, dento-osseous anomalies were found in the family with HNPCC; however, the relationship between HNPCC and dento-osseous anomalies was not clearly established. More studies with a larger case series are necessary to confirm these findings.

## Consent

Written informed consent was obtained from the individuals for publication of this case report and any accompanying images. A copy of the approval (number: 099/2008) by the Ethics Committee of the Faculty of Health Science, University of Brasilia, Brazil, is available for review by the Editor-in-Chief of this journal.

## Competing interests

The authors declare that they have no competing interests.

## Authors’ contributions

FTA, RRG, AFL, ACA, and ENSG designed the study, drafted and revised the manuscript. JBS performed medical examinations and FTA performed oral examinations. ENSG and AFL were responsible for radiological assessment. All authors have read and approved the final manuscript.
